# A Novel Polymethyl Methacrylate Derivative Grafted with Cationic Iridium(III) Complex Units: Synthesis and Application in White Light-Emitting Diodes

**DOI:** 10.3390/polym11030499

**Published:** 2019-03-14

**Authors:** Huaijun Tang, Xueyan Dong, Mingxian Chen, Qiuhong Chen, Mengran Ren, Kaimin Wang, Qiang Zhou, Zhengliang Wang

**Affiliations:** Key Laboratory of Green-Chemistry Materials in University of Yunnan Province, National and Local Joint Engineering Research Center for Green Preparation Technology of Biobased Materials, School of Chemistry & Environment, Yunnan Minzu University, Kunming 650500, China; dong8884567@163.com (X.D.); xsgcmx20110101@163.com (M.C.); chen494300238@163.com (Q.C.); renmengrankevery@sina.com (M.R.); xchow123@126.com (Q.Z.); wzhl629@163.com (Z.W.)

**Keywords:** luminescent polymers, metal-containing polymers, white light-emitting diode, down-conversion luminescent materials, blue GaN chip

## Abstract

A novel orange-yellow-emitting polymethyl methacrylate derivative grafted with cationic iridium(III) complex units was synthesized and used as down-conversion luminescent materials in light-emitting diodes (LEDs). The polymer had a thermal decomposition temperature (*T*_d_) of 275 °C. With the temperature increasing from 20 to 100 °C, its photoluminescent intensity decreased to 76.8% with thermal quenching activation energy (*E*_a_) of 0.2775 eV. A series of LEDs was fabricated by 460 nm blue GaN chips and the polymer blended in silicone at different concentrations. At 4.0 wt %, a cold white LED was obtained, the correlated color temperature (CCT) was 10,050 K, color rendering index (CRI) was 71.2, luminous efficiency (*η*_L_) was 5.3 lm·w^−1^, and Commission Internationale de L’Eclairage (CIE) chromaticity coordinates were (0.30, 0.24). At 5.0 wt.%, the LED emitted neutral white light, its CCT was 4938 K, CRI was 75, *η*_L_ was 13.8 lm·w^−1^, and the CIE value was (0.34, 0.27). At 5.5 wt %, 6.0 wt %, 7.0 wt %, and 8.0 wt %, the LEDs all emitted warm white light; their CCTs were 3446, 3093, 2557, and 2337 K, respectively; their CRIs were 73.6, 71.8, 63.8, and 59.0, respectively; their *η*_L_ were 18.1, 16.3, 14.8, and 13.7 lm·w^−1^, respectively; and their CIE values were (0.36, 0.30), (0.40, 0.35), (0.45, 0.38), and (0.50, 0.42), respectively. At 9.0 wt %, the blue light of GaN chip was completely absorbed by the polymer and only the orange-yellow light of the polymer emitted. The results suggested the polymer was a promising orange-yellow-emitting phosphor candidate for white LEDs, especially for warm white LEDs.

## 1. Introduction

As the new generation solid-state light sources, white light-emitting diodes (LEDs) have some fascinating advantages such as high efficiency, energy-saving, long lifetime, small size, and environmentally friendly properties, and have been widely used in general illumination, full-color displays, liquid crystal display backlights, automotive headlamps, and so on [1,2,3]. At present, white LEDs are mainly fabricated by the combination of ultraviolet or blue chips and down-conversion luminescent materials [1,2,3,4,5,6]; relatively, the blue chips are more commonly used since the high brightness blue GaN chips were successfully contrived in the late 1980s and early 1990s [7,8]. For example, the most commonly commercial white LEDs are just fabricated by the combination of blue GaN chips and yellow-light-emitting Y_3_Al_5_O_12_:Ce^3+^ (YAG:Ce) [3,4,5,6]; moreover, the research and development of many new down-conversion luminescent materials was also based on blue GaN chips [9,10,11].

Mainly owing to high stability and high energy conversion efficiency, many inorganic luminescent materials have been used as phosphors in LEDs [5,6,12]; at the same time, because of some other advantages, such as broad emission spectra, good color regulatability via various organic groups, and hydrophobicity, all kinds of organic luminescent materials also have been widely tried, including small-molecule fluorescent dyes [13,14], luminescent organic metal complexes [15,16,17,18,19], luminescent polymers [20,21], metal-organic framework materials [22,23], and so on. Among them, organic iridium(III) complexes and the polymers containing iridium(III) complex units are important and developed rapidly in the past decade and more, as a result of some good photochemical and photophysical properties, including high efficiency (100% theoretical quantum efficiency) and brightness, excellent color tunability via various ligands, high thermal and photic stability, and so on [24,25,26,27]. Organic iridium(III) complexes have been widely used in many luminescence fields, including organic light-emitting diodes (OLEDs) [24,26,27], light-emitting electrochemical cells (LECs) [25,26,28], chemical sensors [29,30], bioimaging [30,31], and so forth. In the past several years, they also have been used as down-conversion luminescent materials in inorganic LEDs, especially in white LEDs [16,17,18,26,32]. For example, in 2013, a kind of composite luminescent materials formed from yellow-emitting [Ir(ppy)_2_(bpy)]PF_6_ (ppy: 2-phenylpyridine; bpy: 2,2′-bipyridine) and blue-emitting Cd-based metal-organic frameworks (MOF) was successfully used for fabricating high-quality white LEDs [18]. In 2016, some white LEDs using rubber-like polymers containing [Ir(ppy)_2_(tb-bpy)]PF_6_ (tb-bpy: 4,4′-ditert-butyl-2,2′-bipyridine) as down-conversion luminescent materials showed high color quality (CRI > 80), high luminous efficiency (>100 lm·W^-1^), and high stabilities of more than 1000 h (extrapolated 4000 h) under continuous operation conditions [17]. In recent years, a series of cationic iridium (III) complexes had been successfully used in white LEDs by our research group [16,33,34]. Moreover, in 2018, we also used a novel red-emitting cationic iridium(III) coordination polymer in warm white LEDs [32]. In order to explore more such luminescent materials, in this work, a novel polymethyl methacrylate derivative grafted with cationic iridium (III) complex units was synthesized and also used in white LEDs.

## 2. Experimental Section

### 2.1. Materials and Equipments

All chemicals and reagents were purchased from Shanghai Titan Scientific Co. Ltd. (in Shanghai, China) and used without further purification unless otherwise stated. ^1^H NMR spectra were recorded on a Bruker AV400 spectrometer operating at 400 MHz with tetramethylsilane (TMS) as internal standard. Mass spectra (MS) were obtained on a Bruker amaZon SL liquid chromatography mass spectrometer (LC-MS) with an electrospray ionization (ESI) interface using acetonitrile as matrix solvent. Infrared spectra were recorded using a Thermo Scientific Nicolet IS10 Fourier transform infrared (FTIR) spectrometer. Thermogravimetry (TG) was measured on a Netzsch STA449F3 thermal analyzer (NETZSCH-Gerätebau GmbH, Selb, Germany) at a heating rate of 10 °C·min^−1^ under N_2_. Photoluminescence (PL) spectra were obtained on a JobinYvon FL3-21 spectrofluorometer, and the temperature of the solid sample was controlled by a temperature controller (REX-C110, Kaituo Compressor Parts Co. Ltd., Dongguan City, China). Molecular weights and molecular weight distribution (*M*_w_/*M*_n_) values of the polymer were determined by Waters 1515 gel permeation chromatograph (GPC) equipped with a Waters 2414 refractive index detector, calibrated using polystyrenes; tetrahydrofuran (THF) was used as an eluent at a flow rate of 1.0 mL·min^−1^ at 40 °C. 

**Scheme 1:**
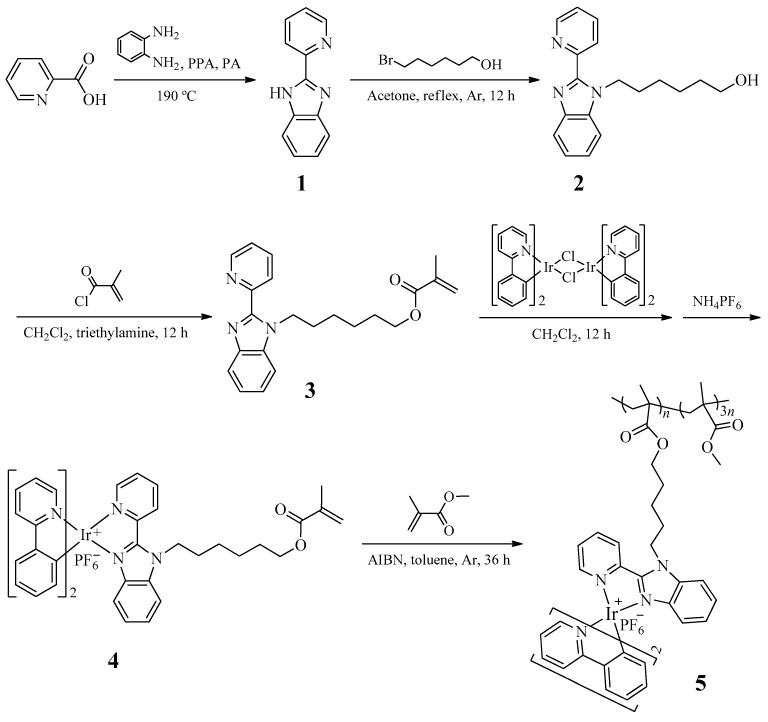
Synthetic route and chemical structure of the polymer.

The synthetic route and chemical structure of the polymer are showed in Scheme 1, experimental details and characterization data are given in the following. 

#### 2.1.1. Synthesis of 2-(pyridin-2-yl)-1H-benzo[d]imidazole (Compound **1**)

Picolinic acid (2.16 g, 20 mmol) and benzene-1,2-diamine (2.46 g, 20 mmol) were added into a mixture of polyphosphoric acid (PPA, 15 mL) and phosphoric acid (PA, 15 mL) and then kept at 190 °C with stirring for 12 h. After being cooled to 80 °C, the reaction mixture was poured into water and then neutralized with Na_2_CO_3_. The resultant precipitate was collected on a filter, then recrystallized from 50% ethanol and filtered. The purified product was obtained as grayish white solid, yield 78.6% (3.06 g). ^1^H NMR (400 MHz, CDCl_3_, ppm), δ: 8.62 (d, 1H, ^3^*J* = 3.76 Hz, pyridine-H), δ: 8.59 (d, 1H, ^3^*J* = 6.32 Hz, pyridine-H), δ: 7.88 (t, 1H, ^3^*J* = 6.08 Hz, pyridine-H), δ: 7.70 (d, 2H, ^3^*J* = 2.24 Hz, phenyl-H), δ: 7.37–7.41 (m, 1H, pyridine-H), δ:7.31–7.35 (m, 2H, phenyl-H).

#### 2.1.2. Synthesis of 6-(2-(pyridin-2-yl)-1H-benzo[d]imidazol-1-yl)hexan-1-ol (Compound **2**)

2-(pyridin-2-yl)-1*H*-benzo[*d*]imidazole (1.95 g, 10 mmol) and KOH (2.81 g, 50 mmol) were added into acetone (35 mL), and then the mixture was kept under argon at room temperature with stirring for 0.5 h. Then, 6-bromohexan-1-ol (1.81 g, 10 mmol) was added into the mixture, after which it was refluxed under argon for 12 h. After being cooled to room temperature, the reaction mixture was poured into water (200 mL), neutralized with Na_2_CO_3_, and then extracted with dichloromethane (3 × 30 mL). The organic phase was washed with brine and dried by anhydrous MgSO_4_. After the solvent was removed, the residue was purified by silica gel column chromatography using dichloromethane and methanol (volume rate, 100:1) as the eluent. Yield 64.6% (1.91 g), colorless oil. ^1^H NMR (400 MHz, DMSO-d_6_, ppm), δ: 8.71–8.75 (m, 1H, pyridine-H), δ: 8.32 (d, 1H, ^3^*J* = 7.96 Hz, pyridine-H), δ: 7.96–8.02 (m, 1H, pyridine-H), δ: 7.73 (d, 1H, ^3^*J* = 7.60 Hz, phenyl-H), δ: 7.65 (d, 1H, ^3^*J* = 7.80 Hz, phenyl-H), δ: 7.48–7.53 (m, 1H, pyridine-H), δ: 7.23–7.34 (m, 2H, phenyl-H), δ: 4.81 (t, 2H, ^3^*J* = 7.40 Hz, −N−CH_2_−), δ: 4.35 (t, 1H, ^3^*J* = 5.08 Hz, −OH), δ: 3.28–3.35 (m, 2H, −CH_2_−O−), δ: 1.75 (t, 2H, ^3^*J* = 6.72 Hz, alkyl-H), δ: 1.22–1.35 (m, 6H, alkyl-H).

#### 2.1.3. Synthesis of 6-(2-(pyridin-2-yl)-1H-benzo[d]imidazol-1-yl)hexyl Methacrylate (Compound **3**)

Under argon atmosphere, triethylamine (0.91 g, 9.0 mmol) was added into a solution of compound **2** (1.77 g, 6.0 mmol) in anhydrous dichloromethane (40 mL). After being cooled to 0 °C, methacryloyl chloride (1.25 g, 12.0 mmol) was dropped into the mixture, and then the reaction mixture was stirred at room temperature for 12 h. After the solvent was removed, the residue was purified by silica gel column chromatography using dichloromethane and acetonitrile (volume rate, 100:1) as the eluent. Yield 87.9% (1.92 g), colorless oil. ^1^H NMR (400 MHz, CDCl3, ppm), δ: 8.67 (d, 1H, ^3^*J* = 4.56 Hz, pyridine-H), δ: 8.40 (d, 1H, ^3^*J* = 8.00 Hz, pyridine-H), δ: 7.90–7.78 (m, 2H, phenyl-H), δ: 7.44 (t, 1H, ^3^*J* = 7.36 Hz, pyridine-H), δ: 7.28–7.36 (m, 3H, pyridine-H and phenyl-H), δ: 6.07 (s, 1H, =CH_2_), δ: 5.53 (s, 1H, =CH_2_), δ: 4.83 (t, 2H, ^3^*J* = 7.60 Hz, −N−CH_2_−), δ: 4.10 (t, 2H, ^3^*J* = 6.6 Hz, −O−CH_2_−), δ: 1.87–1.95 (m, 5H, =C−CH_3_, and alkyl-H), δ:1.64 (t, 2H, ^3^*J* = 6.64 Hz, alkyl-H), δ:1.40 (m, 4H, ^3^*J* = 3.28 Hz, alkyl-H).

#### 2.1.4. Synthesis of the Cationic Iridium(III) Complex (Compound **4**)

The chloro-bridged dimer (ppy)_2_Ir(*μ*-Cl)_2_Ir(ppy)_2_ (ppy: 2-phenylpyridine) was prepared according to the reported procedures [35]. A mixture of (ppy)_2_Ir(*μ*-Cl)_2_Ir(ppy)_2_ (2.37 g, 2.2 mmol) and compound **3** (1.45 g, 4.0 mmol) in dichloromethane (40 mL) was heated at 50 °C with stirring for 24 h in the dark. After being cooled to room temperature, NH_4_PF_6_ (6.52 g, 40.0 mmol) was added and stirred for 0.5 h. After the solvent was removed, the residue was purified by silica gel column chromatography using dichloromethane and acetonitrile (volume rate, 20:1) as the eluent. Yield 54.0% (2.39 g), red powders. ^1^H NMR (400 MHz, CDCl_3_, ppm), δ: 8.61–8.72(m, 1H, ArH), δ: 8.36 (t, 1H, ^3^*J* = 7.84 Hz, ArH), δ: 7.99 (d, 1H, ^3^*J* = 5.16 Hz, ArH), δ: 7.91(d, 1H, ^3^*J* = 8.16 Hz, ArH), δ: 7.83(d, 1H, ^3^*J* = 8.16 Hz, ArH), δ: 7.60–7.77 (m, 5H, ArH), δ: 7.49–7.56 (m, 2H, ArH), 7.35–7.42 (m, 2H, ArH), δ: 6.98–7.09 (m, 4H, ArH), δ: 6.88–6.96 (m, 3H, ArH), δ: 6.40 (d, 1H, ^3^*J* = 7.56 Hz, ArH), δ: 6.31 (t, 2H, ^3^*J* = 8.40 Hz, ArH), δ: 6.06 (s, 1H, =CH_2_), δ: 5.52 (s, 1H, =CH_2_), δ: 4.80–5.0 (m, 2H, −N−CH_2_−), δ: 4.08 (t, 2H, ^3^*J* = 6.52 Hz, −O−CH_2_−), δ: 1.98–2.11 (m, 2H, alkyl-H), δ: 1.90 (s, 3H, =C−CH_3_), δ:1.60–1.67 (m, 2H, alkyl–H), δ:1.40-1.56 (m, 4H, alkyl-H).

#### 2.1.5. Synthesis of the Polymer (Compound **5**)

A mixture of the cationic iridium(III) complex (0.50 g, 0.50 mmol), methyl methacrylate (1.65 g, 16.5 mmol), and 2,2’-azobis(2-methylpropionitrile) (AIBN, 0.080 g, 0.49 mmol) in anhydrous toluene (5 mL) was heated at 60 °C with stirring for 36 h under argon atmosphere. After cooling to room temperature, the resultant yellow solid was filtered and solved in tetrahydrofuran (THF, 5mL), and was then added drop by drop into methanol (100 mL), after which the polymer was separated out and filtered again. Such a dissolution and precipitation process was repeated three times. The purified polymer was dried under vacuum at 60 °C for 24 h. Yield 64.6% (1.39 g), yellow powders. FTIR (KBr, cm^−1^): υ = 3134, 2950, 2026, 1727, 1656, 1602, 1439, 1270, 1238, 1141, 1119, 1068, 1002, 849, 751, 628, 551. The number- and weight-averaged molecular weights (*M*_n_, *M*_w_) of this polymer were estimated by GPC to be 14,007 and 18,229, respectively, with polystyrene as a standard.

### 2.2. Fabrication and Performance Measurements of LEDs

LEDs using the polymer as down-conversion luminescent materials were fabricated and measured. The polymer was blended in silicone at different mass ratios of 4.0, 5.0, 6.0, 7.0, 8.0, and 9.0 wt%. Every mixture of the polymer in silicone was stirred homogeneously and coated onto 460 nm-emitting blue GaN chips until just the reflective cavities were filled up. All LEDs were dried and solidified at 150 °C for 3 h. The LEDs were all operated at 20 mA forward current, their electroluminescent spectra were recorded on a high accurate array spectrometer (HSP6000, HongPu Optoelectronics Technology Co. Ltd., Hangzhou City, China), and their performances were measured by an integrating sphere spectroradiometer system (Everfine PMS-50, Everfine Photo-E-Info Co., Ltd., Hangzhou, China).

## 3. Results and Discussion

### 3.1. Photoluminescence Property

**Figure 1:**
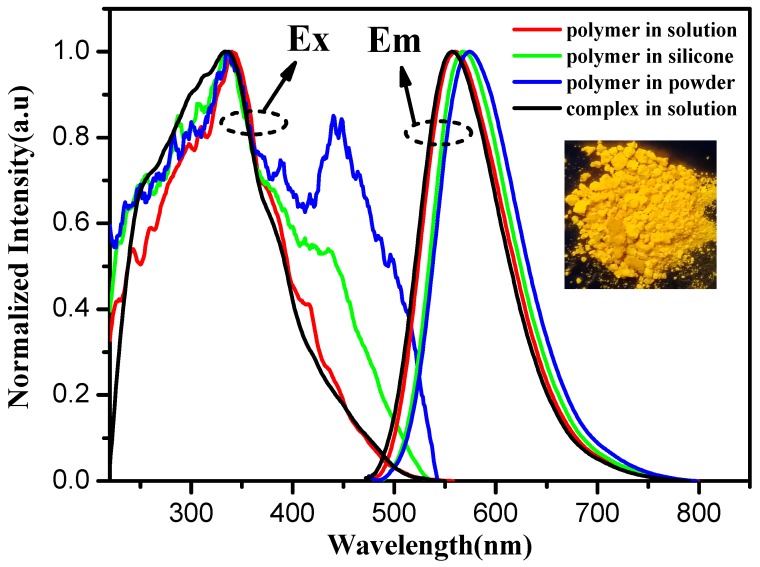
Normalized excitation (Ex) and emission (Em) spectra of the cationic iridium(III) complex (i.e., compound **4**) in CH_2_Cl_2_ solution (1 × 10^−5^ mol·L^−1^), as well as those of the polymer in CH_2_Cl_2_ solution (0.001 wt %), blended in silicone (2.0 wt %, coated on quartz plate), and in powder; the corresponding emission wavelengths were 557, 560, 568, and 574 nm, respectively, and the corresponding excition wavelengths all were 460 nm. Inset: an image of the polymer powder excited by 460 nm blue light.

The normalized photoluminescent excitation (Ex) and emission (Em) spectra of the cationic iridium(III) complex monomer (i.e., compound **4**) in CH_2_Cl_2_ solution (1 × 10^−5^ mol·L^−1^), as well as those of the polymer in CH_2_Cl_2_ solution (0.001 wt %), blended in silicone (2.0 wt %), and in powder are shown in Figure 1. In solution, the emission spectrum of the polymer was very similar to that of the cationic iridium(III) complex monomer, which suggests that luminescence of the polymer originated from the cationic iridium(III) complex units and that the cationic iridium(III) complex units were well dispersed in the polymer chain. Although the excitation spectra of the polymer were measured under three different conditions, they were in the same zone, and all located between 225 nm and 545 nm with the maximum excitation wavelength (λ_ex,max_) of 337 nm. They all covered the blue light zone, which means the polymer can be excited by blue light from GaN chips (415−505 nm, λ_em,max_ = 460 nm). However, in every excitation spectrum, relative to their maximum excitation zone around 337 nm, the relative intensities of the excitation spectra in blue light zone were different; the relative intensity of that in solution was the lowest, in silicone was the second, and in powder was the highest, mainly because of the sequential increasing of molecular interaction and π–π stacking with the increasing concentration of the polymer. Furthermore, mainly because of these reasons, the emission spectra of the polymer in CH_2_Cl_2_ solution, in silicone, and in powder exhibited sequential red shifts; the maximum emission wavelengths were 560 nm, 568 nm, and 574 nm, respectively. Nevertheless, the emission spectra of the polymer under the three different conditions were all mainly located in 485−755 nm, and all of them were orange-yellow light, which contained some red light components. Theoretically, this polymer was beneficial for obtaining warm white light when it was used as luminescent materials in GaN-based white LEDs [34].

### 3.2. Thermal Stability and Thermal Quenching Property

**Figure 2:**
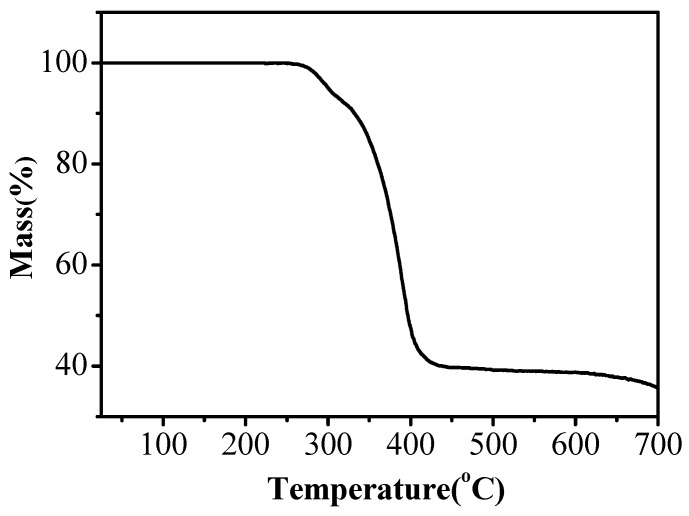
Thermogravimetry (TG) curve of the polymer.

Because of the heat treatment in the device fabrication and exothermic working process of LEDs, it is necessary to investigate the heat resisting property of luminescent materials, mainly including the thermal stability and thermal quenching propery. The thermal stability of the polymer is investigated by thermogravimetry (TG) under N_2_ and the resultant TG curve is shown in Figure 2. The TG curve showed a major mass loss after 275 °C, which means 275 °C was its thermal decomposition temperature (*T*_d_). Such *T*_d_ is enough to meet the heat-resistance requirement because LED devices are usually fabricated and work at a temperature below 150 °C [36].

**Figure 3:**
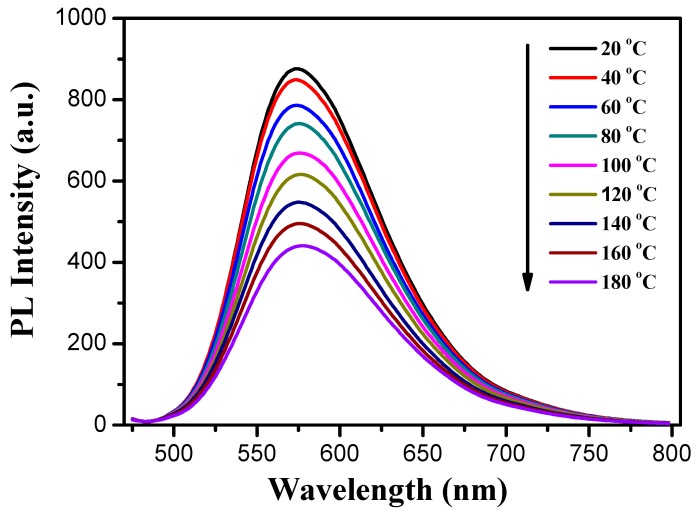
Temperature-dependent photoluminescence (PL) emission spectra of the polymer powders measured with increasing temperature from 20 to 180 °C, λ_ex_ = 460 nm.

The photoluminescent intensity of luminescent materials usually decreases with the increasing temperature, which is known as the thermal quenching property. The photoluminescent spectra of the polymer excited by 460 nm blue light were measured with increasing temperature from 20 to 180 °C and are shown in Figure 3. From 20 to 180 °C, the photoluminescent intensities obviously and sequentially decreased with the increasing temperature. The relative intensities of the maximum emission wavelength (λ_em,max_) at 573 nm were 100% (at 20 °C), 96.8% (at 40 °C), 89.7% (at 60 °C), 84.6% (at 80 °C), 76.8% (at 100 °C), 70.8% (at 120 °C), 62.6% (at 140 °C), 56.6% (at 160 °C), and 50.7% (at 180 °C), respectively. However, the shapes, emission wavelength bands, and λ_em,max_ of these spectra almost did not change, which suggested the light color of the polymer had high stability.

**Figure 4:**
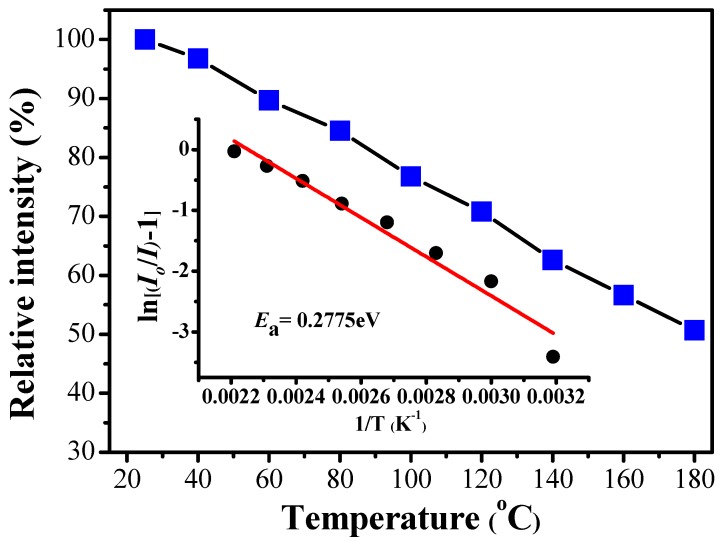
The relative PL intensity of the polymer as a function of temperature. The inset represents the ln[(*I*_o_/*I*) − 1] versus 1/*T* and the calculated activation energy (*E*_a_).

The speed of thermal quenching also can be evaluated and represented by its activation energy (*E*_a_) value; a higher *E*_a_ means slower thermal quenching and higher thermal stability [16,28,37]. The *E*_a_ can be obtained from the variant of Arrhenius equation [28]:

(1) $$\ln(\frac{I_{o}}{I}-1)=(-\frac{E_{a}}{k_{B}})\cdot \frac{1}{T}+\ln A,$$

where *I* and *I*_o_ represent the relative photoluminescent intensity at the experimental temperature (*T*) and room temperature (20 °C, i.e., 293 K), respectively; A is a constant; and *k*_B_ is Boltzmann’s constant (8.617 × 10^−5^ eV·K^−1^). The relative photoluminescent intensities (*I*) of the polymer at the above-mentioned different temperatures (*T*) are shown in Figure 4. The ln[(*I*_o_/*I*) − 1] versus 1/*T* is also shown in an inset in Figure 4. The data were well-fitted, then *E*_a_ can be calculated by the slope value, that is, −(*E*_a_/*k*_B_); which was equal to 0.2775 eV. In comparison with some reported orange-yellow or yellow phosphors in recent years [34,37,38,39], this *E*_a_ value was at an intermediate level, which suggested the polymer had high thermal stability and could be applied in LEDs.

### 3.3. Performances of the Polymer Used in LEDs

**Figure 5:**
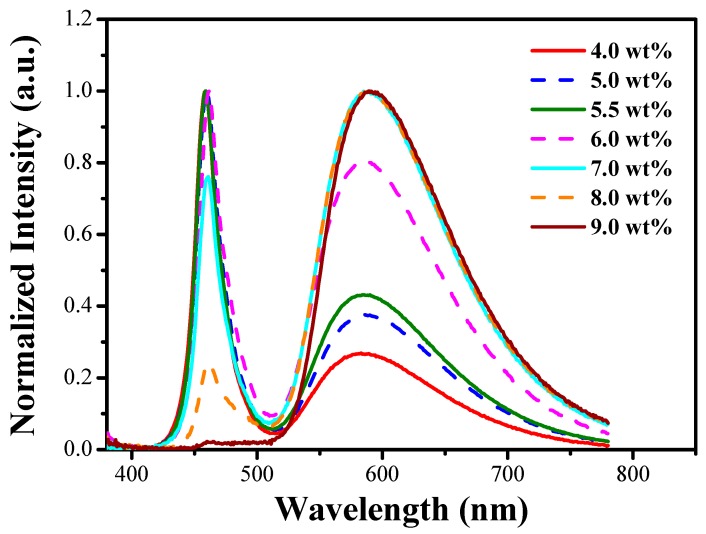
Emission spectra of the blue GaN-based light-emitting diodes (LEDs) using the polymer (*x* wt %, *x* = 4.0, 5.0, 6.0, 7.0, 8.0 and 9.0) as phosphors blended in silicone at different blending concentrations.

In order to investigate the applicability and efficacy of the polymer used as down-conversion luminescent materials in white LEDs, a series of GaN-based LEDs were fabricated using the polymer as down-conversion luminescent materials blended in silicone at different concentrations (*x* wt %, *x* = 4.0, 5.0, 5.5, 6.0, 7.0, 8.0, 9.0). The emission spectra of the LEDs are shown in Figure 5, and the performance data are listed in Table 1.

Almost every spectrum consisted of two emission peaks. The left peaks mainly located at 400~500 nm, and its λ_em, max_ was 460 nm, which can be assigned to the emission of blue GaN chips. The right peaks mainly located at 500~780 nm and were the emission of the polymer. With the increasing of the blending concentrations, the relative emission intensity of the polymer gradually raised and, at the same time, the emission of GaN chips gradually declined and disappeared at 9.0 wt %. Mainly because of the sequential enhancing of molecular interaction and π–π stacking with the increasing of the blending concentrations, the maximum emission wavelengths (λ_em,max_) of the polymer showed a slight red shift, as listed in Table 1, from 4.0 wt % to 9.0 wt %, and the λ_em,max_ changed from 583 nm to 590 nm.

**Figure 6:**
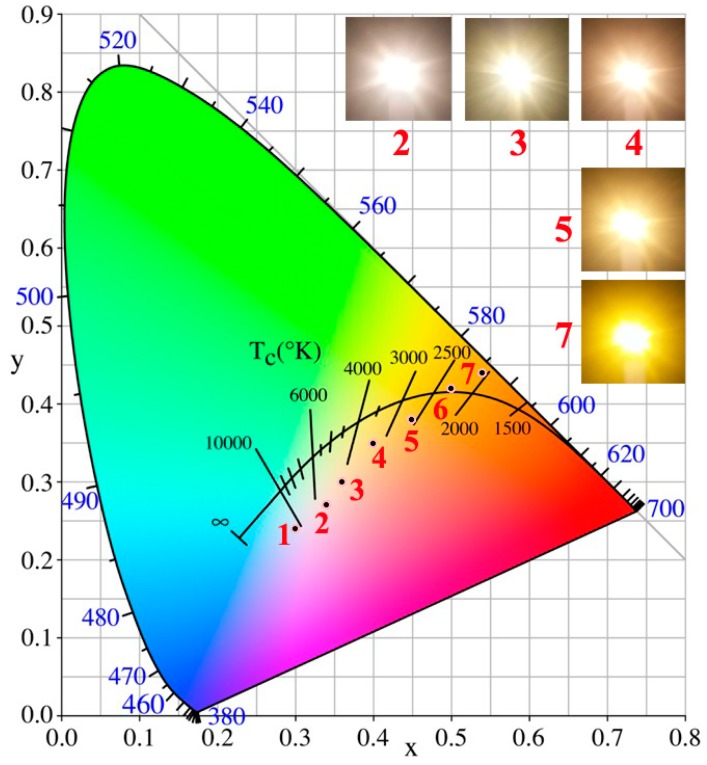
Commission Internationale de L’Eclairage (CIE) chromaticity coordinates of the GaN-based LEDs using the polymer as down-conversion luminescent materials blended in silicone at different concentrations (*x* wt %, *x* = 4.0, 5.0, 5.5, 6.0, 7.0, 8.0, 9.0). Inset: images of the five LEDs, those are, No. 2 (4.0 wt %), No. 3 (5.5 wt %), No. 4 (6.0 wt %), No. 5 (7.0 wt %), and No. 7 (9.0 wt %) in the working state.

As shown in Figure 6, from 4.0 wt % to 9.0 wt %, the Commission Internationale de L’Eclairage (CIE) chromaticity coordinates of the LEDs showed the light color gradually changed from cold white light to neutral white light, to warm white light, and then to orange-yellow light. In detail, at 4.0 wt % (No. 1), the LED emitted cold white light, its correlated color temperature (CCT) was 10,050 K, and its CIE values were (0.30, 0.24). At 5.0 wt % (No. 2), the light color of the LED became neutral white light, its CCT was 4938 K, and its CIE values were (0.34, 0.27). At 5.5 wt % (No. 3), 6.0 wt % (No. 4), 7.0 wt % (No.5), and 8.0 wt % (No. 6), they all were warm white LEDs; their CCTs were 3446 K, 3093 K, 2557 K, and 2337 K, respectively; and their CIE values were (0.36, 0.30), (0.40, 0.35), (0.45, 0.38), and (0.50, 0.42), respectively. At 9.0 wt % (No. 7), because the blue light of the GaN chip was completely absorbed by the polymer, this LED only emitted the orange-yellow light of the polymer, its CCT was 2051 K, and its CIE values were (0.30, 0.24). The working state photographs of No. 2 (neutral white light), No. 3 (warm white light), No. 4 (warm white light), No. 5 (warm white light), and No. 7 (orange-yellow light) are shown in Figure 6 and visually exhibited the above-mentioned changes. As for their color rendering index (CRI), as listed in Table 1, from No. 1 to No. 7, they increased first and then gradually become smaller. At 5.0 wt % (No. 2), the CRI was 75, which was the maximum value and higher than that of YAG:Ce, which was most commonly used in GaN-based white LEDs [16,28,34]. Their luminous efficiencies (*η*_L_) also increased first and then gradually become smaller and reached the maximum value of 18.1 lm·w^−1^ at 5.5 wt % (No. 3). In contrast to similar work in past [16,28,34], especially relative to those of small molecular organic cationic iridium(III) complexes [34], the luminous efficiencies of such LEDs were a bit low, probably be caused by various factors; for example, the doping concentration of the cationic iridium(III) complex units was not optimal, the purity of the polymer needed to be further improved, or the devices needed to be further optimized.

## 4. Conclusions

A novel orange-yellow-emitting polymethyl methacrylate derivative grafted with cationic iridium(III) complex units was synthesized and successfully used as down-conversion luminescent materials in LEDs. The polymer had a thermal decomposition temperature of 275 °C, with the temperature increasing from 20 to 100 °C, and its photoluminescent intensity decreased to 76.8% with thermal quenching *E*_a_ of 0.2775 eV. Under the excition of 460 nm blue GaN chips, the polymer can be used for obtaining cold/neutral/warm white LEDs, especially warm white LEDs. Overall, the best performances of the white LEDs were obtained when the polymer was blended in silicone at 5.5 wt %, the corresponding CRI was 73.6, CCT was 3446 K, the light color was warm white light, CIE values were (0.36, 0.30), and *η*_L_ was 18.1 lm·w^−1^.

## Figures and Tables

**Table 1:** The performance data of GaN-based light-emitting diodes (LEDs) using the polymer as down-conversion luminescent materials blended in silicone at different blending concentrations (*x* wt %, *x* = 4.0, 5.0, 5.5, 6.0, 7.0, 8.0, 9.0). CRI—color rendering index; CCT—correlated color temperature; CIE—Commission Internationale de L’Eclairage.

No. of LEDs	Blending Concentrations (wt %)	Luminous Efficiency (lm·W^−1^)	CRI	CCT (K)	λ_em, max_ (nm)	CIE (*x*, *y*)
1	4.0	5.3	71.2	10050	(460, 583)	(0.30, 0.24)
2	5.0	13.8	75	4938	(460, 586)	(0.34, 0.27)
3	5.5	18.1	73.6	3446	(460, 586)	(0.36, 0.30)
4	6.0	16.3	71.8	3093	(460, 586)	(0.40, 0.35)
5	7.0	14.8	63.8	2557	(460, 586)	(0.45, 0.38)
6	8.0	13.7	59.0	2337	(460, 588)	(0.50, 0.42)
7	9.0	12.4	51.6	2051	(460, 590)	(0.54, 0.44)
